# Building an adaptive trait simulator package to infer parametric diffusion model along phylogenetic tree

**DOI:** 10.1016/j.mex.2020.100978

**Published:** 2020-06-30

**Authors:** Dwueng-Chwuan Jhwueng

**Affiliations:** Department of Statistics, Feng-Chia University, Taichung, Taiwan

**Keywords:** Brownian motion, Cox-ingersoll-ross process, Ornstein-uhlenbeck process, Phylogenetic comparative method, Statistical modeling, Trait evolution

## Abstract

The development of an adaptive trait simulator package for inferring trait evolution along a phylogenetic tree is shown. Stochastic processes of the continuous type are broadly applied to modeling trait evolution when the evolutionary relationship among species and traits of study interest are present. By including several popular stochastic processes, evolutionary information embedded in a dataset can be revealed. The highlights of the method include:

1. The implementation of the popular Cox-Ingersol-Ross process for modeling rate evolution within the package to prevent rates from becoming negative and thus is potentially a useful extension to study adaptive trait evolution in randomly evolved environment.
2. The established trait simulator approach along with approximate Bayesian computation procedure provides a feasible statistical inference without model likelihood.
3. The procedure proposed for trait simulator along phylogenetic tree can be applied to all established models of trait evolution in literature, thus providing users an alternative option to analyze their data.

## Graphical Abstract

**Figure:**
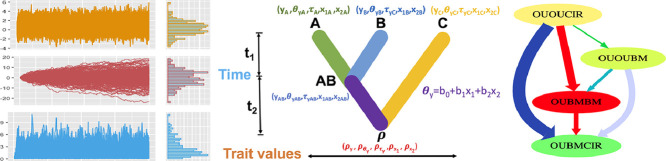

## Specifications table

**Table:** 

Subject Area:	Mathematics, Agricultural and Biological Sciences
More specific subject area:	Stochastic process, Evolutionary biology, Phylogenetic comparative methods
Method name:	Adaptive trait simulator under Ornstein Uhlenbeck process type parametric diffusion model
Name and reference of original method:	Hansen et al. [9], Jhwueng and Maroulas [10,11], Jhwueng [12], Jhwueng (In preparation)
Resource availability:	R package: ouxy at https://CRAN.R-project.org/package=ouxy

## Method detail

### Requirements

 

• Computer system with software environment R for statistical computing and graphics.
• Operating system: Mac OS X 10.0 or higher, Windows XP or higher, Linux distribution: Debian.
• Additional system requirements: none
• Software location: R package
  - Name: ouxy
  - Persistent identifier: https://CRAN.R-project.org/package=ouxy
  - License: GLP-2
  - Publisher: Dwueng-Chwuan Jhwueng
  - Date published: 2020-05-28

## Introduction

The paper focuses on the implementation of methods for adaptive trait evolution in Jhwueng and Maroulas [10], Jhwueng and Maroulas [11] and Jhwueng [12], building from Hansen et al. [9]. Adaptive traits play an important role in survival and reproduction of the organism during the evolutionary process. Any biological trait that helps the organism to survive in its specific environment is an adaptive trait. For example, a snow monkey with thicker (positive) fur would survive better than another monkey with thinner (negative) fur in a very cold environment while the coconut crab evolved lungs to live and breed on land. This motivated the invention of this package that enables feasible inference of trait under the models developed for answering questions from adaptive trait evolution in randomly evolving environment.

Prior work in modeling adaptive trait evolution has been focused on using Gaussian processes such as Brownian motion or Ornstein-Uhlenbeck process where the joint distribution of trait variables in the model follows multivariate normal distribution with analytical mean and variance-covariance structure. This work endeavors to proceed statistical inference on models built by non-Gaussian type process for adaptive trait evolution. Due to the complexity of non-Gaussian processes, the built-in models are often with intractable model likelihood. Inferences under normal approximation to the non-Gaussian type adaptive trait model maybe feasible and efficient for analysis but inferences are remains best and most adequate under the given model.

### Procedure

ouxy, the adaptive trait simulator package created for our purposes, implements models of adaptive trait evolution with an approximate Bayesian computation (ABC) procedure. It was implemented as a library in R [17]. The manual of ouxy can be accessed at R cran with link https://cran.r-project.org/web/packages/ouxy/ouxy.pdf.

While the main function for ouxy is to perform trait simulation along the rooted ultrametric phylogenetic tree under the model of adaptive trait evolution (the OUBMBM model, the OUOUBM model, the OUBMCIR model and the OUOUCIR model, see Additional information - Theory section), it allows for several statistical inferences such as parameter estimation and model selection. The ouxy package contains functions that are separated into three main sections (1) set up the reasonable value for parameter estimation and compute summary statistics from raw input**;** (2) set up appropriate range of prior parameters, perform traits simulation under each model and compute their summary statistics, and (3) conduct statistical inference for parameter estimation and model selection.

Summarized below is the step-by-step process of performing analysis.

1. Prepare your own trait data and a rooted ultrametric phylogenetic tree (an ape tree object).
2. Install the ouxy package in your R environment.
3. Set up the tolerance rate and number of replicates for ABC procedure.
4. Perform the analysis using the main function ouxy.
5. Save the output (posterior parameter estimates and model selection) as desired.

The ABC algorithm presented below adopts the Algorithm 1 in Jhwueng [12].

**Table:** 

**Algorithm**: ouxy ABC rejection algorithm for inferring model of adaptive trait evolution
**input**: A rooted phylogenetic tree T, trait dataset $D=\left(Y,X\right)\;\text{where}\;Y\;\text{is}\;\text{the}\;\text{response}\;\text{trait}\;\text{and}\;X=\left[X_{1},X_{2},...,X_{p}\right]$ is the set of covariate traits, root states $\left(\rho_{Y},\rho_{X}\right)$, prior distribution π(·), hyper parameters for prior $\Theta_{0}$ and tolerate δ
**output**: Posterior samples
1: Compute the summary statistics S(D) for data D
2: **for**j=1,2,⋯,J**do**
3. draw sample $\Theta_{j}$ from prior $\pi (\Theta_{0})$
4. generate trait sample $D_{j}=\left(Y_{j},\;X_{j}\right)$ from $\Theta_{j}$ under a given model M
5. Calculate summary statistics $S(D_{j})$ for simulated data $D_{j}$
6. **end for**
7. Apply ABC **rejection** method using summary statistics S(D) and $S(D_{j}),j=1,2,\cdots ,J$ with a tolerate rate δ
8. **Return** Accepted posterior samples $\Theta_{a}$ where a=1,2,⋯[Jδ] is the nearest integer to Jδ

Note that the distance function in ABC algorithm serves a very important role in accepting the proposed values. Bokma et al. [4] used the Euclidean distance between observed and expected variances as the distance function.

Slater et al. [19] used a partial least squares (PLS) regression transformation of the summary statistics to generate a new, lower dimensional set of summaries prior to computing the distance. The distance function used in Bartoszek et al. ABC algorithm treated the phylogeny and trait data separately. ouxy uses 12 summary statistics (the raw mean, raw variance, raw median, raw skewness, raw kurtosis, the contrast mean, contrast variance, contrast median, contrast skewness, contrast kurtosis [7], Bloomberg's *K* [3] and Pagel's *λ* [15]) and applies the abc rejection method [6] where the Euclidean distance between the summary statistics of raw data and the summary statistics of simulated data is computed, then a specified tolerance rate is used to get the required proportion of points accepted nearest the target values.

Three empirical datasets of bats [1], corals [18] and lizards [14] are included in ouxy. The bat dataset is used as an example.

# First install package 'ouxy'

# install.packages("ouxy")

library(ouxy)

library(phytools)

# use coral data

data(bat)

# call for tree

tree<-bat$tree

# call for traits two covariates: Head height(mm), Head length(mm),

# response variable: Body mass(g).

traitset<-bat$traitset

rownames(traitset)<-tree$tip.label

**Fig. 1:**
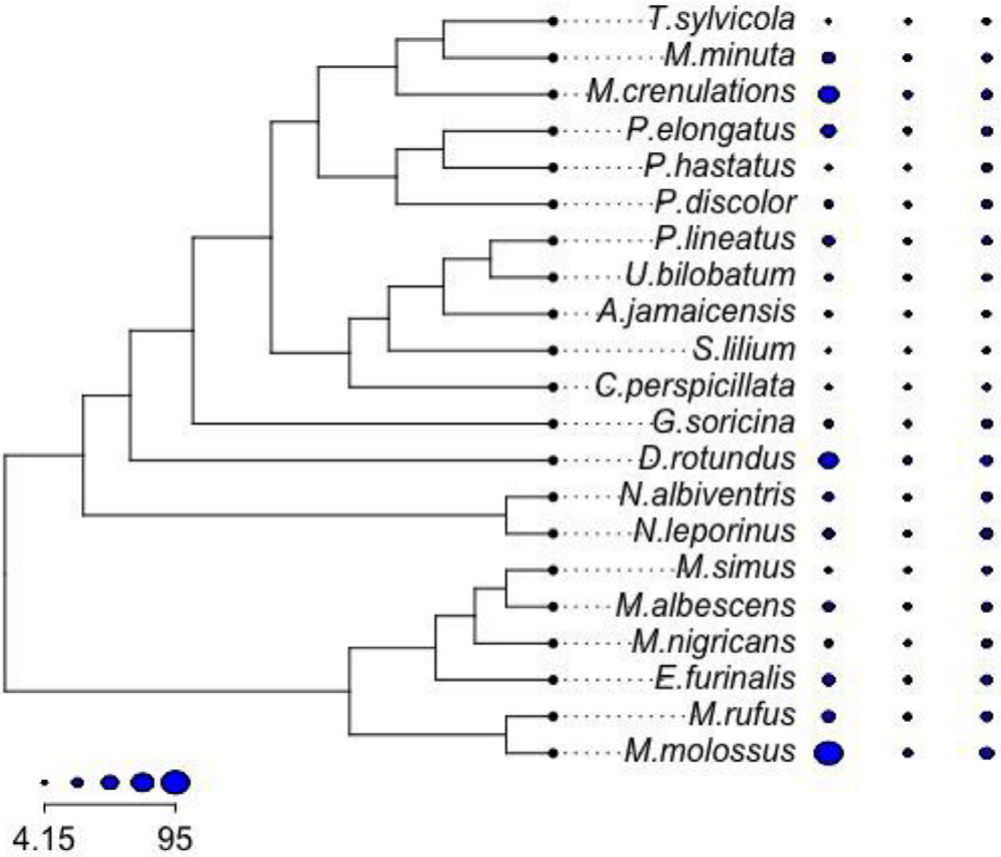
The phylogeny of bats included in ouxy. The trait is shown using blue circles using different sizes. Traits from the left to the right are the response: body mass (g), and two covariates: head height (mm) and head length (mm). Species names are shown in the middle of the plot.

The tree and trait values shown in Fig. 1 can be visualized using the following command

# plot tree and traits

dotTree(tree,traitset,standardize=FALSE,length=6)

All functions developed in package ouxy help performing regular statistical inference and hence are connected to its main function to its functionality and utility. The four built-in models (OUBMBM, OUOUBM, OUBMCIR, OUOUCIR) within this package include procedure that utilize three stochastic processes of continuous type (as the name of the model: BM stands for Brownian motion, OU for Ornstein-Uhlenbeck process and CIR for Cox-Ingersoll-Ross process) and are directly applied to perform comprehensive analysis. The required input are the tree: an ape tree object [16] and trait data set: a data frame where the species name matches the tree tip labels of study interest. For standard procedure of executing statistical inference, the package starts by searching maximum likelihood estimates using OU process (by R function OUprior), then a reasonable range for the prior parameters were generated (by R function HyperParam). Next, the *trait simulator* R functions (oubmbmTrait for the OUBMBM model, ououbmTrait for the OUOUBM model, oubmcirTrait for the OUBMCIR model and ououcirTrait for the OUOUCIR model) generate trait dataset under each model. Then a set of summary statistics are computed (by R function sum.stat).

Finally, posterior samples and model selection under ABC procedure are reported (by R function ouxy). Some procedures such as calculating the summary statistics (by R function sum.stat) or drawing samples for a prior (by one of the R functions oubmbmprior, ououbmprior, oubmcirprior, ououcirprior), can perform independently given the required argument. Hence they can be accessed in the package without calling any other functions.

Among the functions developed in package ouxy, functions oubmbmTrait, ououbmTrait, oubmcirTrait, and ououcirTrait simulate traits along the tree, a key element of the procedure. Conventional methods assume a known statistical distribution for trait variables. Then traits are simulated under the specified distribution. However, the four models (OUBMBM, OUOUBM, OUBMCIR and OUOUCIR) lack an explicit model likelihood, thus the idea is to enable model inference based on approximate Bayesian computation (ABC) technique.

**Fig. 2:**
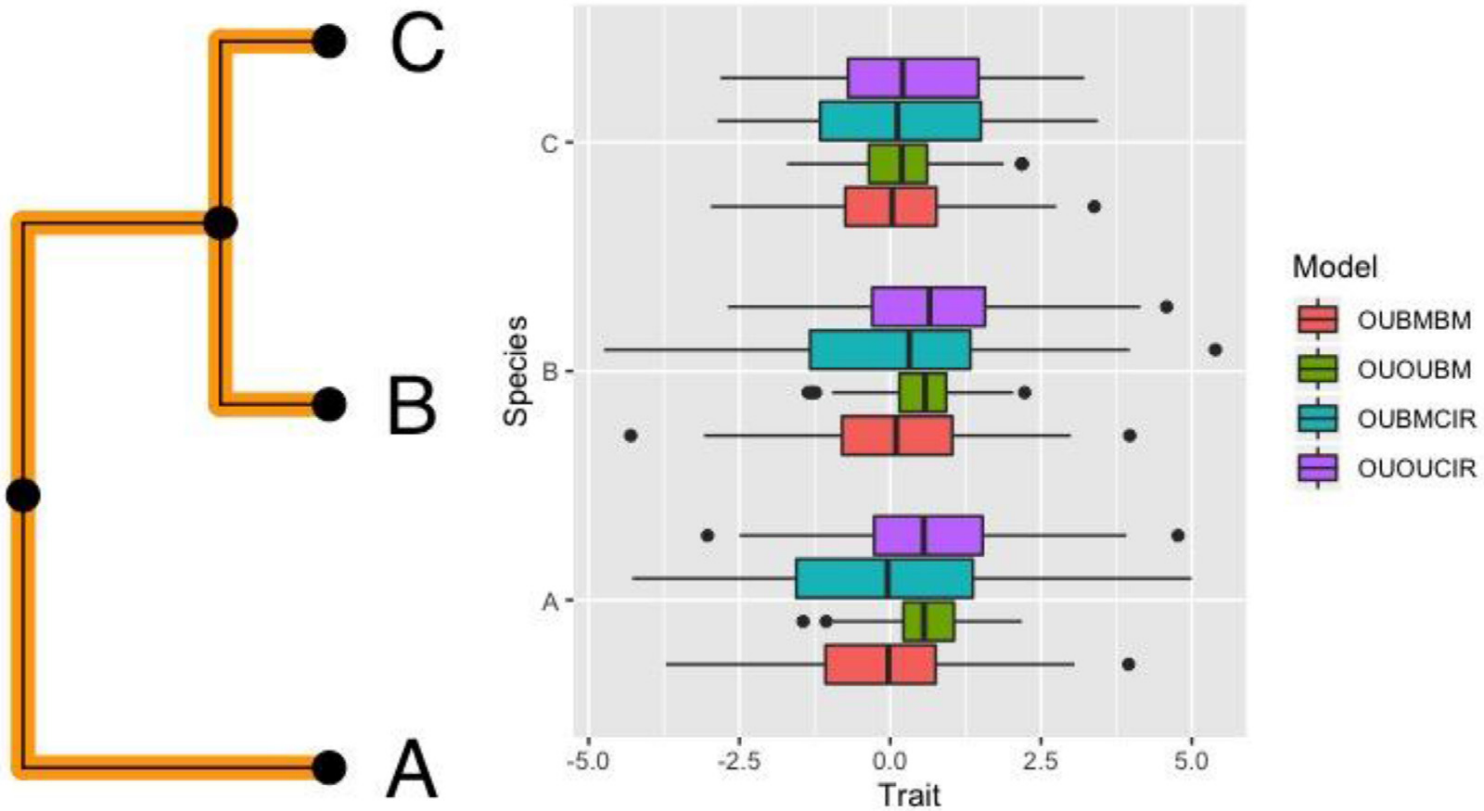
Boxplots for tips simulated under the models of adaptive trait evolution using trait simulator in ouxy. The R script to generate the plots for this simulation can be accessed at online supplement compTraitsim.R

The boxplots for 100 simulated traits using a 3 species phylogenetic tree under each model is shown in Fig. 2.

A particular note is that the representation of trait variable *y_t_* requires solving a system of stochastic integrals that includes the integration of Brownian motion with respect to a Brownian (the OUBMBM model and the OUOUBM model) or the integration of CIR process with respect to a Brownian motion (the OUBMCIR model and the OUOUCIR model). Currently the trait variable *y_t_* is expressed at its most explicit form for each model and is implemented into R functions oubmbmmodel, ououbmmodel, oubmcirmodel and ououcirmodel, repectively.

Once traits are generated under model of interest, samples are drawn from the prior distribution and an ABC procedure is performed for inference. Posterior samples are chosen based on the rejection method using function ouxy where R package abc [6] function abc is used for performing the ABC procedure. Different models are assumed a priori equally likely with same number of simulations.

Bayes factor is defined as a ratio of the posterior model probability of two different models *M_i_* and *M_j_* (i.e. $BF_{ij}=\text{Pr}\left(M_{i}|D\right)/\text{Pr}\left(M_{j}|D\right)$ is used for comparing the fit of models. This is done by using function postpr in R package abc [6] where the posterior model probabilities are estimated using the rejection method. Then the Bayes factor for model *M_i_* over model *M_j_* is computed by the ratio of frequencies of samples from each of these models that are below the threshold.

The following R script performs the analysis.

#perform the analysis using ouxy

#convert trait into ratio scale using a log transform to meet biological assumptions

traitset<-log(traitset)

output<-ouxy(tree=tree,traitset=traitset,tol=0.01,sims= 50000) #It takes a while

#save your file

save.image("bat_analysis.rda")

#save.image("lizard_analysis.rda")

The RData file bat_analysis.rda for the bat data analysis can be directly loaded at http://www.tonyjhwueng.info/ououcir/bat_analysis.rda

load(url("http://www.tonyjhwueng.info/ououcir/bat_analysis.rda"))

For the posterior model probabilities of models and model selection by Bayes factors, execute the following code

output$s.mnlog #based tol*sims = 500 posterior samples among four models

round(output$s.mnlog$rejection$BayesF,4)

**Posterior model probabilities**

**Table:** 

OUBMBM	OUBMCIR	OUOUBM	OUOUCIR
0.5195	0.4295	0.0095	0.0415

**The Bayes factors:**

The Bayes factor *BF*_*ij*_ for the model *i* in the row over the model *j* in the column is shown in the following table.

**Table:** 

*BF*${}_{ij}$	OUBMBM	OUBMCIR	OUOUBM	OUOUCIR
OUBMBM	1.0000	1.2095	54.6842	12.5181
OUBMCIR	0.8268	1.0000	45.2105	10.3494
OUOUBM	0.0183	0.0221	1.0000	0.2289
OUOUCIR	0.0799	0.0966	4.3684	1.0000

For instance, the Bayes factor of the OUBMBM model vs. the OUOUCIR model is shown in the first row and the fourth column in the table with value 12.5181 which is computed by the ratio of the posterior model probability of the OUBMBM model over the posterior model probability of the OUOUCIR model (0.5195÷0.0415 ≈ 12.5181).

Posterior mean for model parameters under each model can be directly accessed by executing the following command

#### Posterior mean of parameters

To generate the table for the posterior mean of parameters, please execute

round(output$table.out,4)

For model parameters

**Table:** 

Model	$\alpha_{y}$	$\alpha_{x}$	$\alpha_{\tau }$	$\theta_{x}$	$\theta_{\tau }$	$\sigma_{x}$	τ
OUBMBM	4.5825					0.7148	6.3979
OUOUBM	1.7844	4.2611		0.7161		0.9532	9.6721
OUBMCIR	4.8636		4.5297		4.5289	0.7224	
OUOUCIR	1.6889	4.1514	3.7677	0.7409	4.8963	0.9144	

For regression parameters

**Table:** 

Model	$b_{0}$	$b_{1}$	$b_{2}$
OUBMBM	-3.9929	2.4837	0.2182
OUOUBM	-4.0451	2.4376	0.0162
OUBMCIR	-4.0590	2.5046	0.1479
OUOUCIR	-3.9936	2.4804	0.1401
GLS	-4.0268	2.5483	0.1958

**Fig. 3:**
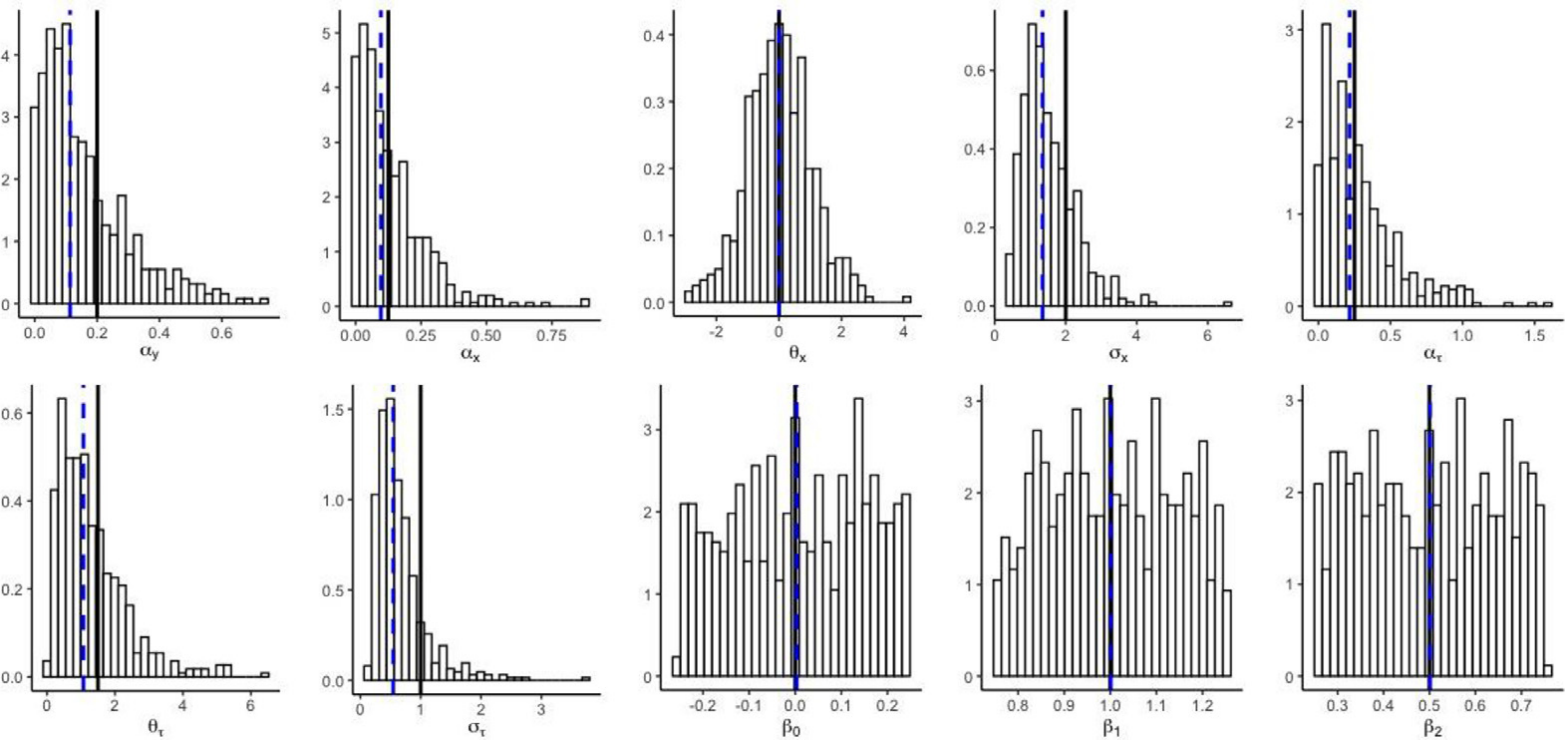
Histogram of posterior parameter estimate for the OUOUCIR model in ouxy. The vertical line in bold in each panel represents the true value and the vertical dashed line is the posterior median. The R script to generate the plots for this simulation can be accessed at online supplemental posthistououcir.r

It is known that statistical inference accounts for the uncertainty. Similar to the simple OU model [2], ABC estimation for the models in Jhwueng [12] results in wide histograms of the estimator (see Fig. 3).

## Additional information

### Theory

ouxy makes use the expression for trait variable *y_t_* that solves the corresponding system of stochastic differential equation for model of adaptive trait evolution. Given a rooted phylogenetic tree with known topology and branch lengths, ouxy makes possible to simulate trait at each node of tree under postorder tree traversal algorithm .

The model starts with an assumption that the trait variable *y_t_* solves the following stochastic differential equation (SDE)

(1) $$dy_{t}=\alpha_{t}^{y}\left(\theta_{t}^{y}-y_{t}\right)\mathrm{dt}+\tau_{t}^{y}dW_{t}^{y},\;t>0,$$

where the deterministic term $\alpha_{t}^{y}\left(\theta_{t}^{y}-y_{t}\right)$ refers to a coefficient that measures the quantity of change in an infinitesimal time *dt* and $\alpha_{t}^{y}$ is the force that pulls *y_t_* back to the optima $\theta_{t}^{y}$; $\tau_{t}^{y}$ is the diffusion coefficient that amplifies/reduces the trait change according to the random changing environment measured by $dW_{t}^{y}$ where $W_{t}^{y}$ is a Wiener process having continuous paths and independent Gaussian increments (i.e. $dW_{t}^{y}∼N\left(0,dt\right)$).

With $\alpha_{t}^{y}=\alpha_{y}$ a constant, direct integration on both side of the Eq. (1) yields to the solution of *y_t_* in Eq. (2)

(2) $$y_{t}=y_{0}+e^{-\alpha_{y}t}\underset{0}{\int^{t}}\alpha_{y}e^{\alpha_{y}s}\theta_{s}^{y}\mathrm{ds}+e^{-\alpha_{y}t}\underset{0}{\int^{t}}\tau_{s}^{y}e^{\alpha_{y}s}dW_{s}^{y}$$

The optimum $\theta_{s}^{y}$ is assumed in a linear relationship with the covariates $x_{k,s},k=1,2,\cdots ,p$ with the representation in Eq. (3)

(3) $$\theta_{s}^{y}=b_{0}+\sum_{k=1}^{p}b_{k}x_{k,s}+\sum_{i=1}^{p}\sum_{j\neq i}^{p}b_{ij}x_{i,s}x_{j,s}$$

where the stochastic variable $x_{k,s},k=1,2,\cdots ,p$ is assumed following either a BM (i.e $dx_{k,s}=\sigma_{x}dW_{s}^{x_{k}}$) or an OU process (i.e. $dx_{k,s}=\alpha_{x}\left(\theta_{x}-x_{k,s}\right)ds+\sigma_{x}dW_{s}^{x_{k}}$) [8]. The rate of evolution $\tau_{s}^{y}$ is either assumed following a Brownian Motion (i.e. $d\tau_{s}^{y}=\tau dW_{s}^{\tau }$) or a CIR process [5] that solves the SDE in Eq. (4)

(4) $$d\tau_{s}^{y}=\alpha_{\tau }\left(\theta_{\tau }-\tau_{s}^{y}\right)\mathrm{ds}+\sigma_{\tau }\sqrt{\tau_{s}^{y}}dW_{s}^{\tau },$$

where *θ_τ_* > 0 is the optimum of $\tau_{s}^{y}$, *α_τ_* > 0 is a constant that pulls $\tau_{s}^{y}$ back to *θ_τ_, σ_τ_* > 0 is the rate of change for $\tau_{s}^{y}$, and $W_{s}^{\tau }$ is a Wiener process.

Details of the derivation for the solution *y_t_* for each model under various assumptions of the corresponding diffusion processes, its optimum parameter $\theta_{s}^{y}$ and rate parameter $\tau_{s}^{y}$ can be accessed in Jhwueng [12].

### Problems encountered

#### Proper approximation to the posterior

Attractive as this model is, it is important to understand the limits of this approach. Below are a few difficulties encountered when developing this package.

Currently, due to the computational requirement for proper approximation to the posterior probabilities of models, it is time consuming to finish the simulation when a sufficiently large of sample is used. In particular, for the model that requires the numerical approximation of the integral of Brownian motion with respect to another Brownian motion or to numerically evaluate the integral of a CIR process random variable with respect to a Brownian motion, drawing sufficiently large trait samples from larger tree (e.g. tree more than 100 taxa) often takes a long running time to finish the analysis.

Moreover, when modeling the rate parameter with CIR process (the OUBMCIR model and the OUOUCIR model), drawing a sample for *y_t_* requires computing a double stochastic integral in Eq. (5)

(5) $$\sigma_{\tau }\int_{0}^{t}e^{-\left(\alpha_{y}+\alpha_{\tau }\right)s}\left(\int_{0}^{s}e^{\alpha_{\tau }u}\sqrt{\tau_{u}}dW_{u}^{\tau }\right)dW_{s}^{y}\;\approx \;\sigma_{\tau }\sum_{i=1}^{m_{2}}\left(e^{-\left(\alpha_{y}+\alpha_{\tau }\right)i*\frac{t}{m_{2}}}\left(\sum_{j=1}^{m_{1}}e^{\alpha_{\tau }j*\frac{s_{i}}{m_{1}}}\sqrt{\tau_{j}}W_{j}^{\tau }\right)W_{i}^{\tau }\right).$$

Conventional theory of numerical analysis suggests finer grids on the time domain should be used to attain more accuracy when computing integrals. However, the stochastic integrals in the left hand side of Eq. (5) has large variation and often produce widely spread trait values when more grids are used. Hence larger uncertainty on the trait value is encountered when more grids are used where more random samples are drawn on each grid. Currently, one grid ($m_{1}=m_{2}=1$) is used to draw a sample from the integral in Eq. (5) implemented in the OUBMCIR and the OUOUCIR models.

Since the models of adaptive trait evolution implement more than one process (OU, BM, CIR) to track the dynamics of a trait along the tree, statistical inference may heavily depend on the simulated data due to the complexity of models. It is also possible that traits simulated from the model lie in a particular region of trait space, in this case one research direction to specify the region in trait space may start by using the trait simulator developed here.

#### Extension to non-linear optimal regression

In evolutionary biology, regression analysis is broadly applied to model the relationship between dependent variables and their covariates. However, across the diversity of life these functional relationships will vary. A possible extension is to implement nonlinear optimal regresssion connecting the functional relationship between response $\theta_{t}^{y}$ and covariates *x_t_* [13].

For instance, given a Brownian motion (or an Ornstein Uhlenbeck process) covariate *x_t_*, the exponential relationship $\theta_{t}^{y}=b_{0}+b_{1}\text{exp}\left(b_{2}x_{t}\right)$ converts $\theta_{t}^{y}$ to a geometric Brownian motion (or a geometric Ornstein Uhlenbeck process) stochastic variable; while $\theta_{t}^{y}=b_{0}+b_{1}{\left(x_{t}-b_{2}\right)}^{2}$ transforms $\theta_{t}^{y}$ into a squared Brownian motion (or a Cox-Ingersoll-Ross process) stochastic variable. However, for an Ornstein Uhlenbeck process covariate. (i.e. $x_{t}=\theta_{x}+\text{exp}\left(-\alpha_{x}t\right)\left(x_{0}-\theta_{x}+\sigma_{x}\int_{0}^{t}\text{exp}\left(\alpha_{x}s\right)dW_{s}^{x}\right)$), *y_t_* in Eq. (2) is analytically intractable with unknown finite dimensional distribution. Moreover, in computing the covariance between two tips that involves the geometric Ornstein Uhlenbeck process, it remains computational challenge to get a reliable estimate for the term in Eq. (6)

(6) $$E\left[\int_{0}^{t}{\left(\text{exp}\left(\alpha_{y}s+\beta_{3}x_{s}\right)ds\right)}^{2}\right]=E\left[\int_{0}^{t}{\left(\text{exp}(\alpha_{y}s+\beta_{3}\left(\theta_{x}+\text{exp}\left(-\alpha_{x}s\right)\left(x_{0}-\theta_{x}+\sigma_{x}\int_{0}^{s}\text{exp}\left(\alpha_{x}u\right)dW_{u}^{x}\right)\right)ds\right)}^{2}\right]$$

Currently a better numerical scheme is required to approximate the quantity in Eq. (6).

Note that a popular package pcmabc [2] available on CRAN offers the same functionality as ouxy except for a wider class of models, including a trait dependent speciation one. While pcmabc allows arbitrary class of Markov process and requires users to specify the drift coefficient and diffusion coefficient in the diffusion model, ouxy focus on expanding the Ornstein-Uhlenbeck based processes with non-Gaussian process (CIR) on the rate of evolution and functional optimal regression where the optimal of the dependent variable depends on another stochastic covariates. pcmabc. ouxy offers a convenient and an alternative option for users to choose suitable models best to describe their empirical data.

## Funding

This research was financially supported by grant of the 10.13039/501100004663Taiwan Ministry of Science and Technology 108-2118-M-035-001 and was assisted by attendance as a Short-term Visitor at the National Institute for Mathematical and Biological Synthesis, an Institute supported by the 10.13039/100000001National Science Foundation through NSF Award # DBI-1300426, with additional support from The University of Tennessee, Knoxville.

## Declaration of Competing Interest

None declared.

## Appendix Supplementary materials

Image, application 1

Image, application 2

Image, application 3

## References

[1] Aguirre L.F., Herrel A., van Damme R., Matthysen E. (2002). Ecomorphological analysis of trophic niche partitioning in a tropical savannah bat community. Proc. R. Soc. Lond. B: Biol. Sci..

[2] Bartoszek K., Liò P. (2019). Modelling trait dependent speciation with approximate Bayesian computation. Acta Physica Polonica B Proc. Suppl..

[3] Blomberg S.P, Garland T., Ives A.R. (2003). Testing for phylogenetic signal in comparative data: behavioral traits are more labile. Evolution.

[4] Bokma F. (2010). Time, species, and separating their effects on trait variance in clades. Systemat. Biol..

[5] Cox J.C., Ingersoll J.E., Ross S.A. (1985). A theory of the term structure of interest rates. Econometrica.

[6] Csilléry K., François O., Blum M.GB (2012). Abc: An R Package for Approximate Bayesian Computation (Abc). Methods Ecol. Evol..

[7] Felsenstein J. (1985). Phylogeny and the comparative method. Am. Naturalist.

[8] Hansen T.F. (1997). Stabilizing selection and the comparative analysis of adaptation. Evolution.

[9] T. Hansen, J. Pienaar, and S. Orzack. 2008. “A comparative method for studying adaptation to a randomly evolving environment” 62 (July): 1965–77. 10.1111/j.1558-5646.2008.00412.x 18452574

[10] Jhwueng D.-C., Maroulas V. (2014). Phylogenetic Ornstein-Uhlenbeck regression curves. Stat. Probab. Lett..

[11] Jhwueng D.-C., Maroulas Vasileios (2016). Adaptive trait evolution in random environment. J. Appl. Stat..

[12] Jhwueng D.-C. (2020). Modeling rate of adaptive trait evolution using Cox–Ingersoll–Ross Process: an approximate Bayesian computation approach. Comput. Stat. Data Anal..

[13] D.-C. Jhwueng, Phylogenetic non-linear optimal regression for adaptive trait evolution. 2020. In Preparation. 10.3390/e23020218 PMC7916804 33579023

[14] Molina-Borja M, Rodriguez-Dominguez MA (2004). Evolution of Biometric and Life-History Traits in Lizards (Gallotia) from the Canary Islands. J. Zool. Systemat. Evol. Res..

[15] Pagel M. (1999). Inferring the historical patterns of biological evolution. Nature.

[16] Paradis E., Schliep K. (2018). Ape 5.0: An environment for modern phylogenetics and evolutionary analyses in R. Bioinformatics.

[17] R Core Team (2019). R: A Language and Environment for Statistical Computing. https://www.R-project.org/.

[18] Sanchez J.A., Lasker H.R. (2003). Patterns of morphological integration in marine modular organisms: supra-module organization in branching octocoral colonies. Proc.: Biol. Sci..

[19] Slater Graham J, Harmon Luke J, Wegmann Daniel, Joyce Paul, Revell Liam J, Alfaro Michael E (2012). Fitting models of continuous trait evolution to incompletely sampled comparative data using approximate Bayesian computation. Evol.: Int. J. Organ. Evol..

