# Supplementary material for: Building an adaptive trait simulator package to infer parametric diffusion model along phylogenetic tree
Source: MethodsX. 2020 Jun 30;7:100978. doi: 10.1016/j.mex.2020.100978 (PMC7341453; doi:10.1016/j.mex.2020.100978)
Supplement: Supplementary file 2 [file mmc2.pdf]

Package ‘ouxy’

May 21, 2020

Type Package

Title Model of Adaptive Trait Evolution

Version 1.1

Date 2019-09-15

Author Dwueng-Chwuan Jhwueng

Maintainer Dwueng-Chwuan Jhwueng <dcjhwueng@fcu.edu.tw>

Description Performs statistical inference on the models of adaptive trait evolution under approximate Bayesian computation. This can simulate traits from four models, compute trait data summary statistics. Parameters are estimated under Approximate Bayesian Computation, model selection as well as posterior parameter mean will be reported. Users need to enter a comparative dataset and a phylogenetic tree.

License GPL (>=2)

RoxygenNote 7.0.2

Depends R (>= 3.5.0)

Imports stats, ape, coda, Sim.DiffProc, MCMCpack, abc, phytools, nlme, TreeSim, adephylo, maps, geiger, EasyABC, utils

Suggests testthat

BugReports https://github.com/djhwueng/ououcir/issues

Encoding UTF-8

NeedsCompilation no

R topics documented:

bat 2
coral 3
HyperParam 3
lizard 4
oubmbmmodel 4
oubmbmprior 6
oubmbmTrait 7
oubmcirmodel 8
oubmcirprior 9
oubmcirTrait 10
oubmmodel 11
oubmprior 12

oubmTrait . . . 13
ougbmmodel . . . 14
ougbmprior . . . 15
ougbmTrait . . . 16
ougoumodel . . . 17
ougouprior . . . 18
ougouTrait . . . 19
ououbmmodel . . . 20
ououbmprior . . . 21
ououbmTrait . . . 22
ououcirmodel . . . 23
ououcirprior . . . 24
ououcirTrait . . . 25
ououmodel . . . 26
ououprior . . . 27
ououTrait . . . 28
OUprior . . . 29
ouqbmmodel . . . 30
ouqbmprior . . . 31
ouqbmTrait . . . 32
ouqoumodel . . . 33
ouqouprior . . . 34
ouqouTrait . . . 35
ouxy . . . 36
regboundfcn . . . 37
sumstat . . . 38

Index **40**

`bat` *Bat datset*

Description

A dataset containing a phylogenetic tree and trait data for bat species

Usage

```
bat
```

Format

A list of two items

`tree` the phylogenetic tree object

`traitset` a data frame of trait data

References

Aguirre, Luis F., et al. "Ecomorphological analysis of trophic niche partitioning in a tropical savannah bat community." Proceedings of the Royal Society of London. Series B: Biological Sciences 269.1497 (2002): 1271-1278.

`coral` *Coral datset*

Description

A dataset containing a phylogenetic tree and trait data for coral species

Usage

```
coral
```

Format

A list of two items

`tree` the phylogenetic tree object

`traitset` a data frame of trait data

References

Sanchez, Juan Armando, and Howard R. Lasker. "Patterns of morphological integration in marine modular organisms: supra-module organization in branching octocoral colonies." Proceedings of the Royal Society of London. Series B: Biological Sciences 270.1528 (2003): 2039-2044.

`HyperParam` *The range of parameters*

Description

Set up range for parameters for next step

Usage

```
HyperParam(tree = tree, traitset = traitset)
```

Arguments

`tree`	An ape: tree object stored in phylo format
`traitset`	a dataframe that contains 3 traits

Details

Function `OUprior` is called to compute the model estimate, then return the range for parameter estimate for next step analysis. The range is set to 3 times larger/smaller than the parameter estimates. Function `regboundfcn` is called to get the bound of regression parameter. The ancestral value (root) is computed for each traits in order to used for simulation in the four functions `oubmbmTrait`,`ououbmTrait`, `oubmcirTrait` and `ououcirTrait`.

Value

A list of vectors of sample of model parameters, regression parameter and ancestral values.

Examples

```
## using coral dataset (running time more > 5 sec)

data(coral)
tree<-coral$tree
traitset<-coral$traitset
HyperParam(tree=tree,traitset=traitset)
```

`lizard` *Lizard datset*

Description

A dataset containing a phylogenetic tree and trait data for lizard species

Usage

```
lizard
```

Format

A list of two items

`tree` the phylogenetic tree object

`traitset` a data frame of trait data

References

Molina-Borja, M., & Rodriguez-Dominguez, M. A. (2004). Evolution of biometric and life-history traits in lizards (Gallotia) from the Canary Islands. Journal of Zoological Systematics and Evolutionary Research, 42(1), 44-53.

`oubmbmmodel` *Simulate traits under OUBMBM model given a set of parameters*

Description

Simulate traits under OUBMBM model given a set of model parameters, regression parameters, tree and ancestral values.

Usage

```
oubmbmmodel(model.params, reg.params, root = root, tree = tree)
```

Arguments

`model.params`	A vector of model parameters including alpha.y: force parameter of response trait, sigmasq.x: rate parameter of covariate and tau: rate parameter of response trait
`reg.params`	A vector of regression paramters including b0: intercept parameter of regression, b1: slope parameter of first covariate, b2: slope paramter of the second covariate
`root`	A vector of numerical values for root of species estimated from `OUprior` estimated from `OUprior`
`tree`	An ape: tree object stored in phylo format

Details

The model requires user to input model parameters $\alpha_y, \sigma_x^2, \tau$ and regression parameters b_0, b_1, b_2, a tree object, trait dataset (one response, two covariate), ancestral values(root) which is estimated by BM or OU model from `geiger`. Then the algorithm starts from the root and apply the tree traversal algorithm to simulate trait on each node according the OUOUBM dynamics.

Value

Returns the trait vectors $Y = (y_1, y_2, \cdots, y_n)', X_1 = (x_{1,1}, x_{1.2}, \cdots, x_{1,n})', X_2 = (x_{2,1}, x_{2,2}, \cdots, x_{2,n})'$ simulated from the model.

References

1. Jhwueng, D-C. (2019) Statistical modeling for adaptive trait evolution. Under review.
2. Jhwueng, D-C., and Vasileios Maroulas. "Adaptive trait evolution in random environment." Journal of Applied Statistics 43.12 (2016): 2310-2324.
3. Hansen, Thomas F., Jason Pienaar, and Steven Hecht Orzack. "A comparative method for studying adaptation to a randomly evolving environment." Evolution: International Journal of Organic Evolution 62.8 (2008): 1965-1977.

Examples

```
library(ape)
tree<-rcoal(5)
tree<-reorder(tree,"postorder")
root<-list(y.ou.sigmsq=1 ,y.ou.root=0, x1.ou.root=0, x2.ou.root=0,x1.bm.root=0, x2.bm.root=0)
model.params<-c(0.5,1,0.2)
names(model.params)<-c("alpha.y","sigmasq.x","tau")
reg.params<-c(0,1,1)
names(reg.params)<-c("b0","b1","b2")
oubmbmmodel(model.params,reg.params,root=root,tree=tree)
```

oubmbmprior *Draw prior samples for OUBMBM model*

Description

Simulate sample for parameters in OUBMBM model given a set of hyper parameters

Usage

```
oubmbmprior(
  prior.model.params = prior.model.params,
  prior.reg.params = prior.reg.params
)
```

Arguments

`prior.model.params`
: A vectors of hyper parameters for model parameter containing (alpha.y.min, alpha.y.max) for alpha.y, (sigmasq.x.min, sigmasq.x.max) for sigmasq.x, (tau.y.min, tau.max) for rate parameter of tau

`prior.reg.params`
: A vector of hyper paramter for regression parameters. (b0.min,b0.max) for b0, (b1.min,b1.max) for b1, (b2.min,b2.max) for b2

Details

The function requires user to input hyper parameters for $\alpha_y, \sigma_x^2, \tau$ and hyper parameters for regression parameters b_0, b_1, b_2 from uniform distribution with its minimum and maximum values.

Value

Returns the samples of model parameters and regression parameter

Examples

```
prior.model.params<-c(0,3,0,3,0,1)

names(prior.model.params)<-c("alpha.y.min","alpha.y.max",

"tau.min","tau.max","sigmasq.x.min","sigmasq.x.max")

prior.reg.params<-c(-3, 3, -3, 3, -3, 3)
names(prior.reg.params)<-c("b0.min", "b0.max", "b1.min", "b1.max", "b2.min", "b2.max")
oubmbmprior(prior.model.params=prior.model.params,prior.reg.params=prior.reg.params)
```

oubmbmTrait *Parameter samples and summary statistics*

Description

Draw sample for parameters, simulate trait and compute the summary statistics for OUBMBM model

Usage

```
oubmbmTrait(tree = tree, traitset = traitset, sims = sims)
```

Arguments

tree	An ape: tree object stored in phylo format
traitset	a dataframe that contains 3 traits
sims	number of trait replicate

Details

Given tree, trait sets, function HyperParam is called to yield the range of parameters, then function oubmbmprior is called to draw sample for parameter, then the function oubmbmmodel is applied to simulate traits through post order tree traversal algorithm, finally the summary statistics is computed by function sumstat.

Value

A list of vectors containing a dataframe of summary statistics, and a dataframe of parameter samples

Examples

```
## using bat dataset (running time more > 5 sec)

data(bat)
tree<-bat$tree
traitset<-bat$traitset
sims<-10
oubmbmTrait(tree=tree,traitset=traitset,sims=sims)
```

`oubmcirmodel` *Simulate traits under OUBMCIR model given a set of parameters*

Description

Simulate traits under OUBMCIR model given a set of model parameters, regression parameters, tree and ancestral values.

Usage

```
oubmcirmodel(model.params, reg.params, root = root, tree = tree)
```

Arguments

`model.params`	A vector of model parameters including alpha.y: force parameter of response trait, sigmasq.x: rate parameter of covariate, alpha.tau: force parameter of rate, theta.tau optimum parameter of rate, sigmasq.tau: rate parameter of rate
`reg.params`	A vector of regression paramters including b0: intercept parameter of regression, b1: slope parameter of first covariate, b2: slope paramter of the second covariate
`root`	A vector of numerical values for root of species estimated from `OUprior`
`tree`	An ape: tree object stored in phylo format

Details

The model requires user to input model parameters $\alpha_y, \sigma^2_x, \alpha_\tau, \theta_\tau, \sigma^2_\tau$ and regression parameters b_0, b_1, b_2, a tree object, trait dataset (one response, two covariate), ancestral values(root) which is estimated by BM or OU model from `geiger`. Then the algorithm starts from the root and apply the tree traversal algorithm to simulate trait on each node according the OUOUBM dynamics.

Value

Returns the trait vectors $Y = (y_1, y_2, \cdots, y_n)', X_1 = (x_{1,1}, x_{1.2}, \cdots, x_{1,n})', X_2 = (x_{2,1}, x_{2,2}, \cdots, x_{2,n})'$ simulated from the model.

References

1. Jhwueng, D-C. (2019) Statistical modeling for adaptive trait evolution. Under review.
2. Jhwueng, D-C., and Vasileios Maroulas. "Adaptive trait evolution in random environment." Journal of Applied Statistics 43.12 (2016): 2310-2324.
3. Hansen, Thomas F., Jason Pienaar, and Steven Hecht Orzack. "A comparative method for studying adaptation to a randomly evolving environment." Evolution: International Journal of Organic Evolution 62.8 (2008): 1965-1977.

Examples

```
library(ape)
tree<-rcoal(5)
tree<-reorder(tree,"postorder")
root<-list(y.ou.sigmsq=1 ,y.ou.root=0, x1.ou.root=0, x2.ou.root=0,x1.bm.root=0, x2.bm.root=0)
model.params<-c(0.5,0.25,0.3,1,0.2)
names(model.params)<-c("alpha.y","alpha.x","theta.x","sigmasq.x","sigmasq.tau")
reg.params<-c(0,1,1)
names(reg.params)<-c("b0","b1","b2")
oubmcirmodel(model.params,reg.params,root=root,tree=tree)
```

`oubmcirprior` *Draw prior samples for OUBMCIR model*

Description

Simulate sample for parameters in OUBMCIR model given a set of hyper parameters

Usage

```
oubmcirprior(
  prior.model.params = prior.model.params,
  prior.reg.params = prior.reg.params
)
```

Arguments

`prior.model.params`
: A vectors of hyper parameters for model parameter containing (alpha.y.min, alpha.y.max) for alpha.y, (sigmasq.x.min, sigmasq.x.max) for sigmasq.x, (alpha.tau.min, alpha.tau.max) for alpha.tau, (theta.tau.min, theta.tau.max) for theta_tau, (sigmasq.tau.min, sigmasq.tau.max) for rate parameter of tau

`prior.reg.params`
: A vector of hyper paramter for regression parameters. (b0.min,b0.max) for b0, (b1.min,b1.max) for b1, (b2.min,b2.max) for b2

Details

The function requires user to input hyper parameters for $\alpha_y, \sigma^2_x, \alpha_\tau, \theta_\tau, \sigma^2_\tau$ and hyper parameters for regression parameters b_0, b_1, b_2 from uniform distribution with its minimum and maximum values.

Value

Returns the samples of model parameters and regression parameter

Examples

```
prior.model.params<-c(0,3,0,3,0,1,0,3,0,2,0,1.5)


names(prior.model.params)<-c(

"alpha.y.min","alpha.y.max","sigmasq.x.min","sigmasq.x.max",

"alpha.tau.min","alpha.tau.max","theta.tau.min","theta.tau.max",

"sigmasq.tau.min","sigmasq.tau.max")
prior.reg.params<-c(-3, 3, -3, 3, -3, 3)
names(prior.reg.params)<-c("b0.min", "b0.max", "b1.min", "b1.max", "b2.min", "b2.max")
oubmcirprior(prior.model.params=prior.model.params,prior.reg.params=prior.reg.params)
```

---

oubmcirTrait *Parameter samples and summary statistics*

Description

Draw sample for parameters, simulate trait and compute the summary statistics for OUBMCIR model

Usage

```
oubmcirTrait(tree = tree, traitset = traitset, sims = sims)
```

Arguments

`tree`	An ape: tree object stored in phylo format
`traitset`	a dataframe that contains 3 traits
`sims`	number of trait replicate

Details

Given tree, trait sets, function `HyperParam` is called to yield the range of parameters, then function `oubmbmprior` is called to draw sample for parameter, then the function `oubmbmmodel` is applied to simulate traits through post order tree traversal algorithm, finally the summary statistics is computed by function `sumstat`.

Value

A list of vectors containing a dataframe of summary statistics, and a dataframe of parameter samples

Examples

```
## using coral dataset (running time more > 5 sec)

data(coral)
tree<-coral$tree
traitset<-coral$traitset
sims<-10
oubmcirTrait(tree=tree,traitset=traitset,sims=sims)
```

`oubmmodel` *Simulate traits under OUBM model given a set of parameters*

Description

Simulate traits under OUBM model given a set of model parameters, regression parameters, tree and ancestral values.

Usage

```
oubmmodel(model.params, reg.params, root = root, tree = tree)
```

Arguments

`model.params`	A vector of model parameters including alpha.y: force parameter of response trait, sigmasq.x: rate parameter of covariate and tau: rate parameter of response trait
`reg.params`	A vector of regression paramters including b0: intercept parameter of regression, b1: slope parameter of first covariate, b2: slope paramter of the second covariate
`root`	A vector of numerical values for root of species estimated from `OUprior` estimated from `OUprior`
`tree`	An ape: tree object stored in phylo format

Details

The model requires user to input model parameters $\alpha_y, \sigma^2_x, \tau$ and regression parameters b_0, b_1, b_2, a tree object, trait dataset (one response, two covariate), ancestral values(root) which is estimated by BM or OU model from `geiger`. Then the algorithm starts from the root and apply the tree traversal algorithm to simulate trait on each node according the OUBM dynamics.

Value

Returns the trait vectors $Y = (y_1, y_2, \cdots, y_n)', X_1 = (x_{1,1}, x_{1.2}, \cdots, x_{1,n})', X_2 = (x_{2,1}, x_{2,2}, \cdots, x_{2,n})'$ simulated from the model.

References

1. Jhwueng, D. C. (2020). Modeling rate of adaptive trait evolution using Cox–Ingersoll–Ross process: An Approximate Bayesian Computation approach. Computational Statistics & Data Analysis, 145, 106924.
2. Jhwueng, D-C., and Vasileios Maroulas. "Adaptive trait evolution in random environment." Journal of Applied Statistics 43.12 (2016): 2310-2324.
3. Hansen, Thomas F., Jason Pienaar, and Steven Hecht Orzack. "A comparative method for studying adaptation to a randomly evolving environment." Evolution: International Journal of Organic Evolution 62.8 (2008): 1965-1977.

Examples

```
library(ape)
tree<-rcoal(5)
tree<-reorder(tree,"postorder")
root<-list(y.ou.sigmsq=1 ,y.ou.root=0, x1.ou.root=0, x2.ou.root=0,x1.bm.root=0, x2.bm.root=0)
model.params<-c(0.5,1,0.2)
names(model.params)<-c("alpha.y","sigmasq.x","tau")
reg.params<-c(0,1,1)
names(reg.params)<-c("b0","b1","b2")
oubmmodel(model.params,reg.params,root=root,tree=tree)
```

---

oubmprior *Draw prior samples for OUBM model*

Description

Simulate sample for parameters in OUBM model given a set of hyper parameters

Usage

```
oubmprior(
  prior.model.params = prior.model.params,
  prior.reg.params = prior.reg.params
)
```

Arguments

`prior.model.params`
: A vectors of hyper parameters for model parameter containing (alpha.y.min, alpha.y.max) for alpha.y, (sigmasq.x.min, sigmasq.x.max) for sigmasq.x, (tau.y.min, tau.max) for rate parameter of tau

`prior.reg.params`
: A vector of hyper paramter for regression parameters. (b0.min,b0.max) for b0, (b1.min,b1.max) for b1, (b2.min,b2.max) for b2

Details

The function requires user to input hyper parameters for $\alpha_y, \sigma^2_x, \tau$ and hyper parameters for regression parameters b_0, b_1, b_2 from uniform distribution with its minimum and maximum values.

Value

Returns the samples of model parameters and regression parameter

Examples

```
prior.model.params<-c(0,3,0,3,0,1)

names(prior.model.params)<-c("alpha.y.min","alpha.y.max",

"tau.min","tau.max","sigmasq.x.min","sigmasq.x.max")

prior.reg.params<-c(-3, 3, -3, 3, -3, 3)
names(prior.reg.params)<-c("b0.min", "b0.max", "b1.min", "b1.max", "b2.min", "b2.max")
oubmprior(prior.model.params=prior.model.params,prior.reg.params=prior.reg.params)
```

---

oubmTrait *Parameter samples and summary statistics*

Description

Draw sample for parameters, simulate trait and compute the summary statistics for OUBM model

Usage

```
oubmTrait(tree = tree, traitset = traitset, sims = sims)
```

Arguments

tree	An ape: tree object stored in phylo format
traitset	a dataframe that contains 3 traits
sims	number of trait replicate

Details

Given tree, trait sets, function `HyperParam` is called to yield the range of parameters, then function `oubmprior` is called to draw sample for parameter, then the function `oubmmodel` is applied to simulate traits through post order tree traversal algorithm, finally the summary statistics is computed by function `sumstat`.

Value

A list of vectors containing a dataframe of summary statistics, and a dataframe of parameter samples

Examples

```
## using bat dataset (running time more > 5 sec)

data(bat)
tree<-bat$tree
traitset<-bat$traitset
sims<-10
oubmTrait(tree=tree,traitset=traitset,sims=sims)
```

---

ougbmmodel *Simulate traits under ougbm model given a set of parameters*

Description

Simulate traits under ougbm model given a set of model parameters, regression parameters, tree and ancestral values.

Usage

```
ougbmmodel(model.params, reg.params, root = root, tree = tree)
```

Arguments

`model.params`	A vector of model parameters including alpha.y: force parameter of response trait, sigmasq.x: rate parameter of covariate and tau: rate parameter of response trait
`reg.params`	A vector of regression paramters including b0: intercept parameter of regression, b1: slope parameter of first covariate, b2: slope paramter of the second covariate
`root`	A vector of numerical values for root of species estimated from `OUprior` estimated from `OUprior`
`tree`	An ape: tree object stored in phylo format

Details

The model requires user to input model parameters $\alpha_y, \sigma_x^2, \tau$ and regression parameters b_0, b_1, b_2, a tree object, trait dataset (one response, two covariate), ancestral values(root) which is estimated by BM or OU model from `geiger`. Then the algorithm starts from the root and apply the tree traversal algorithm to simulate trait on each node according the ougbm dynamics.

Value

Returns the trait vectors $Y = (y_1, y_2, \cdots, y_n)', X_1 = (x_{1,1}, x_{1.2}, \cdots, x_{1,n})', X_2 = (x_{2,1}, x_{2,2}, \cdots, x_{2,n})'$ simulated from the model.

References

1. Jhwueng, D. C. (2020). Modeling rate of adaptive trait evolution using Cox–Ingersoll–Ross process: An Approximate Bayesian Computation approach. Computational Statistics & Data Analysis, 145, 106924.
2. Jhwueng, D-C., and Vasileios Maroulas. "Adaptive trait evolution in random environment." Journal of Applied Statistics 43.12 (2016): 2310-2324.
3. Hansen, Thomas F., Jason Pienaar, and Steven Hecht Orzack. "A comparative method for studying adaptation to a randomly evolving environment." Evolution: International Journal of Organic Evolution 62.8 (2008): 1965-1977.

Examples

```
library(ape)
tree<-rcoal(5)
tree<-reorder(tree,"postorder")
root<-list(y.ou.sigmsq=1 ,y.ou.root=0, x1.ou.root=0, x2.ou.root=0,x1.bm.root=0, x2.bm.root=0)
model.params<-c(0.5,1,0.2)
names(model.params)<-c("alpha.y","sigmasq.x","tau")
reg.params<-c(0,1,1)
names(reg.params)<-c("b0","b1","b2")
ougbmmodel(model.params,reg.params,root=root,tree=tree)
```

---

ougbmprior — *Draw prior samples for ougbm model*

Description

Simulate sample for parameters in ougbm model given a set of hyper parameters

Usage

```
ougbmprior(
  prior.model.params = prior.model.params,
  prior.reg.params = prior.reg.params
)
```

Arguments

`prior.model.params`
: A vectors of hyper parameters for model parameter containing (alpha.y.min, alpha.y.max) for alpha.y, (sigmasq.x.min, sigmasq.x.max) for sigmasq.x, (tau.y.min, tau.max) for rate parameter of tau

`prior.reg.params`
: A vector of hyper paramter for regression parameters. (b0.min,b0.max) for b0, (b1.min,b1.max) for b1, (b2.min,b2.max) for b2

Details

The function requires user to input hyper parameters for $\alpha_y, \sigma_x^2, \tau$ and hyper parameters for regression parameters b_0, b_1, b_2 from uniform distribution with its minimum and maximum values.

Value

Returns the samples of model parameters and regression parameter

Examples

```
prior.model.params<-c(0,3,0,3,0,1)

names(prior.model.params)<-c("alpha.y.min","alpha.y.max",

"tau.min","tau.max","sigmasq.x.min","sigmasq.x.max")

prior.reg.params<-c(-3, 3, -3, 3, -3, 3)
names(prior.reg.params)<-c("b0.min", "b0.max", "b1.min", "b1.max", "b2.min", "b2.max")
ougbmprior(prior.model.params=prior.model.params,prior.reg.params=prior.reg.params)
```

---

ougbmTrait *Parameter samples and summary statistics*

Description

Draw sample for parameters, simulate trait and compute the summary statistics for ougbm model

Usage

```
ougbmTrait(tree = tree, traitset = traitset, sims = sims)
```

Arguments

tree	An ape: tree object stored in phylo format
traitset	a dataframe that contains 3 traits
sims	number of trait replicate

Details

Given tree, trait sets, function HyperParam is called to yield the range of parameters, then function ougbmprior is called to draw sample for parameter, then the function ougbmmodel is applied to simulate traits through post order tree traversal algorithm, finally the summary statistics is computed by function sumstat.

Value

A list of vectors containing a dataframe of summary statistics, and a dataframe of parameter samples

Examples

```
## using bat dataset (running time more > 5 sec)

data(bat)
tree<-bat$tree
traitset<-bat$traitset
sims<-10
ougbmTrait(tree=tree,traitset=traitset,sims=sims)
```

---

ougoumodel — *Simulate traits under ougou model given a set of parameters*

Description

Simulate traits under ougou model given a set of model parameters, regression parameters, tree and ancestral values.

Usage

```
ougoumodel(model.params, reg.params, root = root, tree = tree)
```

Arguments

`model.params`	A vector of model parameters including alpha.y: force parameter of response trait, alpha.x: force parameter of covariate, theta.x: optimum parameter of covariate, sigmasq.x: rate parameter of covariate and tau rate parameter of response trait
`reg.params`	A vector of regression paramters including b0: intercept parameter of regression, b1: slope parameter of first covariate, b2: slope paramter of the second covariate
`root`	A vector of numerical values for root of species estimated from `OUprior`
`tree`	An ape: tree object stored in phylo format

Details

The model requires user to input model parameters $\alpha_y, \alpha_x, \theta_x, \sigma_x^2, \tau$ and regression parameters b_0, b_1, b_2, a tree object, trait dataset (one response, two covariate), ancestral values(root) which is estimated by BM or OU model from `geiger`. Then the algorithm starts from the root and apply the tree traversal algorithm to simulate trait on each node according the ougou dynamics.

Value

Returns the trait vectors $Y = (y_1, y_2, \cdots, y_n)', X_1 = (x_{1,1}, x_{1.2}, \cdots, x_{1,n})', X_2 = (x_{2,1}, x_{2,2}, \cdots, x_{2,n})'$ simulated from the model.

References

1. Jhwueng, D-C. (2019) Statistical modeling for adaptive trait evolution. Under review.
2. Jhwueng, D-C., and Vasileios Maroulas. "Adaptive trait evolution in random environment." Journal of Applied Statistics 43.12 (2016): 2310-2324.
3. Hansen, Thomas F., Jason Pienaar, and Steven Hecht Orzack. "A comparative method for studying adaptation to a randomly evolving environment." Evolution: International Journal of Organic Evolution 62.8 (2008): 1965-1977.

Examples

```
library(ape)
tree<-rcoal(5)
tree<-reorder(tree,"postorder")
root<-list(y.ou.sigmsq=1 ,y.ou.root=0, x1.ou.root=0, x2.ou.root=0,x1.bm.root=0, x2.bm.root=0)
model.params<-c(0.5,0.25,0,1,0.2)
names(model.params)<-c("alpha.y","alpha.x","theta.x","sigmasq.x","tau")
reg.params<-c(0,1,1)
names(reg.params)<-c("b0","b1","b2")
ougoumodel(model.params,reg.params,root=root,tree=tree)
```

---

ougouprior *Draw prior samples for ougou model*

Description

Simulate sample for parameters in ougou model given a set of hyper parameters

Usage

```
ougouprior(
  prior.model.params = prior.model.params,
  prior.reg.params = prior.reg.params
)
```

Arguments

`prior.model.params`
: A vectors of hyper parameters for model parameter containing (alpha.y.min, alpha.y.max) for alpha.y, (alpha.x.min, alpha.x.max) for alpha.x,(theta.y.min, theta.y.max) for theta.y, (sigmasq.x.min, sigmasq.x.max) for sigmasq.x, (tau.y.min, tau.max) for rate parameter of tau

`prior.reg.params`
: A vector of hyper paramter for regression parameters. (b0.min,b0.max) for b0, (b1.min,b1.max) for b1, (b2.min,b2.max) for b2

Details

The function requires user to input hyper parameters for $\alpha_y, \alpha_x, \theta_x, \sigma_x^2, \tau$ and hyper parameters for regression parameters b_0, b_1, b_2 from uniform distribution with its minimum and maximum values.

Value

Returns the samples of model parameters and regression parameter

Examples

```
prior.model.params<-c(0,3,0,3,-5,5,0,1,0,2)
names(prior.model.params)<-c(

"alpha.y.min","alpha.y.max","alpha.x.min","alpha.x.max",

"theta.x.min","theta.x.max","sigmasq.x.min","sigmasq.x.max",

"tau.min","tau.max")
prior.reg.params<-c(-3, 3, -3, 3, -3, 3)
names(prior.reg.params)<-c("b0.min", "b0.max", "b1.min", "b1.max", "b2.min", "b2.max")
ougouprior(prior.model.params=prior.model.params,prior.reg.params=prior.reg.params)
```

---

ougouTrait — *Parameter samples and summary statistics*

Description

Draw sample for parameters, simulate trait and compute the summary statistics for ougou model

Usage

```
ougouTrait(tree = tree, traitset = traitset, sims = sims)
```

Arguments

`tree`	An ape: tree object stored in phylo format
`traitset`	a dataframe that contains 3 traits
`sims`	number of trait replicate

Details

Given tree, trait sets, function `HyperParam` is called to yield the range of parameters, then function `oubmbmprior` is called to draw sample for parameter, then the function `oubmbmmodel` is applied to simulate traits through post order tree traversal algorithm, finally the summary statistics is computed by function `sumstat`.

Value

A list of vectors containing a dataframe of summary statistics, and a dataframe of parameter samples

Examples

```
## using lizard dataset (running time more > 5 sec)

data(lizard)
tree<-lizard$tree
traitset<-lizard$traitset
sims<-10
ougouTrait(tree=tree,traitset=traitset,sims=sims)
```

`ououbmmodel` *Simulate traits under OUOUBM model given a set of parameters*

Description

Simulate traits under OUOUBM model given a set of model parameters, regression parameters, tree and ancestral values.

Usage

```
ououbmmodel(model.params, reg.params, root = root, tree = tree)
```

Arguments

`model.params`	A vector of model parameters including alpha.y: force parameter of response trait, alpha.x: force parameter of covariate, theta.x: optimum parameter of covariate, sigmasq.x: rate parameter of covariate and tau rate parameter of response trait
`reg.params`	A vector of regression paramters including b0: intercept parameter of regression, b1: slope parameter of first covariate, b2: slope paramter of the second covariate
`root`	A vector of numerical values for root of species estimated from `OUprior`
`tree`	An ape: tree object stored in phylo format

Details

The model requires user to input model parameters $\alpha_y, \alpha_x, \theta_x, \sigma^2_x, \tau$ and regression parameters b_0, b_1, b_2, a tree object, trait dataset (one response, two covariate), ancestral values(root) which is estimated by BM or OU model from `geiger`. Then the algorithm starts from the root and apply the tree traversal algorithm to simulate trait on each node according the OUOUBM dynamics.

Value

Returns the trait vectors $Y = (y_1, y_2, \cdots, y_n)', X_1 = (x_{1,1}, x_{1.2}, \cdots, x_{1,n})', X_2 = (x_{2,1}, x_{2,2}, \cdots, x_{2,n})'$ simulated from the model.

References

1. Jhwueng, D-C. (2019) Statistical modeling for adaptive trait evolution. Under review.
2. Jhwueng, D-C., and Vasileios Maroulas. "Adaptive trait evolution in random environment." Journal of Applied Statistics 43.12 (2016): 2310-2324.
3. Hansen, Thomas F., Jason Pienaar, and Steven Hecht Orzack. "A comparative method for studying adaptation to a randomly evolving environment." Evolution: International Journal of Organic Evolution 62.8 (2008): 1965-1977.

Examples

```
library(ape)
tree<-rcoal(5)
tree<-reorder(tree,"postorder")
root<-list(y.ou.sigmsq=1 ,y.ou.root=0, x1.ou.root=0, x2.ou.root=0,x1.bm.root=0, x2.bm.root=0)
model.params<-c(0.5,0.25,0,1,0.2)
names(model.params)<-c("alpha.y","alpha.x","theta.x","sigmasq.x","tau")
reg.params<-c(0,1,1)
names(reg.params)<-c("b0","b1","b2")
ououbmmodel(model.params,reg.params,root=root,tree=tree)
```

`ououbmprior` *Draw prior samples for OUOUBM model*

Description

Simulate sample for parameters in OUOUBM model given a set of hyper parameters

Usage

```
ououbmprior(
  prior.model.params = prior.model.params,
  prior.reg.params = prior.reg.params
)
```

Arguments

`prior.model.params`
: A vectors of hyper parameters for model parameter containing (alpha.y.min, alpha.y.max) for alpha.y, (alpha.x.min, alpha.x.max) for alpha.x,(theta.y.min, theta.y.max) for theta.y, (sigmasq.x.min, sigmasq.x.max) for sigmasq.x, (tau.y.min, tau.max) for rate parameter of tau

`prior.reg.params`
: A vector of hyper paramter for regression parameters. (b0.min,b0.max) for b0, (b1.min,b1.max) for b1, (b2.min,b2.max) for b2

Details

The function requires user to input hyper parameters for $\alpha_y, \alpha_x, \theta_x, \sigma_x^2, \tau$ and hyper parameters for regression parameters b_0, b_1, b_2 from uniform distribution with its minimum and maximum values.

Value

Returns the samples of model parameters and regression parameter

Examples

```
prior.model.params<-c(0,3,0,3,-5,5,0,1,0,2)
names(prior.model.params)<-c(

"alpha.y.min","alpha.y.max","alpha.x.min","alpha.x.max",

"theta.x.min","theta.x.max","sigmasq.x.min","sigmasq.x.max",

"tau.min","tau.max")
prior.reg.params<-c(-3, 3, -3, 3, -3, 3)
names(prior.reg.params)<-c("b0.min", "b0.max", "b1.min", "b1.max", "b2.min", "b2.max")
ououbmprior(prior.model.params=prior.model.params,prior.reg.params=prior.reg.params)
```

`ououbmTrait` *Parameter samples and summary statistics*

Description

Draw sample for parameters, simulate trait and compute the summary statistics for OUOUBM model

Usage

```
ououbmTrait(tree = tree, traitset = traitset, sims = sims)
```

Arguments

`tree`	An ape: tree object stored in phylo format
`traitset`	a dataframe that contains 3 traits
`sims`	number of trait replicate

Details

Given tree, trait sets, function `HyperParam` is called to yield the range of parameters, then function `oubmbmprior` is called to draw sample for parameter, then the function `oubmbmmodel` is applied to simulate traits through post order tree traversal algorithm, finally the summary statistics is computed by function `sumstat`.

Value

A list of vectors containing a dataframe of summary statistics, and a dataframe of parameter samples

Examples

```
## using lizard dataset (running time more > 5 sec)

data(lizard)
tree<-lizard$tree
traitset<-lizard$traitset
sims<-10
ououbmTrait(tree=tree,traitset=traitset,sims=sims)
```

`ououcirmodel` *Simulate traits under OUBMCIR model given a set of parameters*

Description

Simulate traits under OUBMCIR model given a set of model parameters, regression parameters, tree and ancestral values.

Usage

```
ououcirmodel(model.params, reg.params, root = root, tree = tree)
```

Arguments

`model.params`	A vector of model parameters including alpha.y: force parameter of response trait, alpha.x: force parameter of covariate, theta.x" optimum parameter of covariate, sigmasq.x: rate parameter of covariate, alpha.tau: force parameter of rate, theta.tau optimum parameter of rate, sigmasq.tau: rate parameter of rate
`reg.params`	A vector of regression paramters including b0: intercept parameter of regression, b1: slope parameter of first covariate, b2: slope paramter of the second covariate
`root`	A vector of numerical values for root of species estimated from `OUprior`
`tree`	An ape: tree object stored in phylo format

Details

The model requires user to input model parameters $\alpha_y, \alpha_x, \theta_x, \sigma^2_x, \alpha_\tau, \theta_\tau, \sigma^2_\tau$ and regression parameters b_0, b_1, b_2, a tree object, trait dataset (one response, two covariate), ancestral values(root) which is estimated by BM or OU model from `geiger`. Then the algorithm starts from the root and apply the tree traversal algorithm to simulate trait on each node according the OUOUBM dynamics.

Value

Returns the trait vectors $Y = (y_1, y_2, \cdots, y_n)', X_1 = (x_{1,1}, x_{1.2}, \cdots, x_{1,n})', X_2 = (x_{2,1}, x_{2,2}, \cdots, x_{2,n})'$ simulated from the model.

References

1. Jhwueng, D-C. (2019) Statistical modeling for adaptive trait evolution. Under review.
2. Jhwueng, D-C., and Vasileios Maroulas. "Adaptive trait evolution in random environment." Journal of Applied Statistics 43.12 (2016): 2310-2324.
3. Hansen, Thomas F., Jason Pienaar, and Steven Hecht Orzack. "A comparative method for studying adaptation to a randomly evolving environment." Evolution: International Journal of Organic Evolution 62.8 (2008): 1965-1977.

Examples

```
library(ape)
tree<-rcoal(5)
tree<-reorder(tree,"postorder")
root<-list(y.ou.sigmsq=1 ,y.ou.root=0, x1.ou.root=0, x2.ou.root=0,x1.bm.root=0, x2.bm.root=0)
model.params<-c(0.5,0.25,0,1,0.125,0.15,0.2)
names(model.params)<-c("alpha.y","alpha.x","theta.x","sigmasq.x"

,"alpha.tau","theta.tau","sigmasq.tau")
reg.params<-c(0,1,1)
names(reg.params)<-c("b0","b1","b2")
ououcirmodel(model.params,reg.params,root=root,tree=tree)
```

`ououcirprior` *Draw prior samples for OUOUCIR model*

Description

Simulate sample for parameters in OUOUCIR model given a set of hyper parameters

Usage

```
ououcirprior(
  prior.model.params = prior.model.params,
  prior.reg.params = prior.reg.params
)
```

Arguments

`prior.model.params`
: A vectors of hyper parameters for model parameter containing (alpha.y.min, alpha.y.max) for alpha.y, (alpha.x.min, alpha.x.max) for alpha.x, (theta.x.min, theta.x.max) for theta.x, (sigmasq.x.min, sigmasq.x.max) for sigmasq.x, (alpha.tau.min, alpha.tau.max) for alpha.tau, (theta.tau.min, theta.tau.max) for theta_tau, (sigmasq.tau.min, sigmasq.tau.max) for rate parameter of tau

`prior.reg.params`
: A vector of hyper paramter for regression parameters. (b0.min,b0.max) for b0, (b1.min,b1.max) for b1, (b2.min,b2.max) for b2

Details

The function requires user to input hyper parameters for $\alpha_y, \alpha_x, \theta_x, \sigma_x^2, \alpha_\tau, \theta_\tau, \sigma_\tau^2$ and hyper parameters for regression parameters b_0, b_1, b_2 from uniform distribution with its minimum and maximum values.

Value

Returns the samples of model parameters and regression parameter

Examples

```
prior.model.params<-c(0,3,0,3,-2,2,0,1,0,3,0,2,0,1.5,0,1)


names(prior.model.params)<-c(

"alpha.y.min","alpha.y.max","alpha.x.min","alpha.x.max",

"theta.x.min","theta.x.max","sigmasq.x.min","sigmasq.x.max",

"alpha.tau.min","alpha.tau.max","theta.tau.min","theta.tau.max",

"sigmasq.tau.min","sigmasq.tau.max")
prior.reg.params<-c(-3, 3, -3, 3, -3, 3)
names(prior.reg.params)<-c("b0.min", "b0.max", "b1.min", "b1.max", "b2.min", "b2.max")
ououcirprior(prior.model.params=prior.model.params,prior.reg.params=prior.reg.params)
```

---

ououcirTrait *Parameter samples and summary statistics*

Description

Draw sample for parameters, simulate trait and compute the summary statistics for OUOUCIR model

Usage

```
ououcirTrait(tree = tree, traitset = traitset, sims = sims)
```

Arguments

`tree`	An ape: tree object stored in phylo format
`traitset`	a dataframe that contains 3 traits
`sims`	number of trait replicate

Details

Given tree, trait sets, function `HyperParam` is called to yield the range of parameters, then function `oubmbmprior` is called to draw sample for parameter, then the function `oubmbmmodel` is applied to simulate traits through post order tree traversal algorithm, finally the summary statistics is computed by function `sumstat`.

Value

A list of vectors containing a dataframe of summary statistics, and a dataframe of parameter samples

Examples

```
## using bat dataset (running time more > 5 sec)

data(bat)
tree<-bat$tree
traitset<-bat$traitset
sims<-10
ououcirTrait(tree=tree,traitset=traitset,sims=sims)
```

---

ououmodel *Simulate traits under OUOU model given a set of parameters*

Description

Simulate traits under OUOU model given a set of model parameters, regression parameters, tree and ancestral values.

Usage

```
ououmodel(model.params, reg.params, root = root, tree = tree)
```

Arguments

`model.params`	A vector of model parameters including alpha.y: force parameter of response trait, alpha.x: force parameter of covariate, theta.x: optimum parameter of covariate, sigmasq.x: rate parameter of covariate and tau rate parameter of response trait
`reg.params`	A vector of regression paramters including b0: intercept parameter of regression, b1: slope parameter of first covariate, b2: slope paramter of the second covariate
`root`	A vector of numerical values for root of species estimated from `OUprior`
`tree`	An ape: tree object stored in phylo format

Details

The model requires user to input model parameters $\alpha_y, \alpha_x, \theta_x, \sigma_x^2, \tau$ and regression parameters b_0, b_1, b_2, a tree object, trait dataset (one response, two covariate), ancestral values(root) which is estimated by BM or OU model from `geiger`. Then the algorithm starts from the root and apply the tree traversal algorithm to simulate trait on each node according the OUOU dynamics.

Value

Returns the trait vectors $Y = (y_1, y_2, \cdots, y_n)', X_1 = (x_{1,1}, x_{1.2}, \cdots, x_{1,n})', X_2 = (x_{2,1}, x_{2,2}, \cdots, x_{2,n})'$ simulated from the model.

References

1. Jhwueng, D-C. (2019) Statistical modeling for adaptive trait evolution. Under review.
2. Jhwueng, D-C., and Vasileios Maroulas. "Adaptive trait evolution in random environment." Journal of Applied Statistics 43.12 (2016): 2310-2324.
3. Hansen, Thomas F., Jason Pienaar, and Steven Hecht Orzack. "A comparative method for studying adaptation to a randomly evolving environment." Evolution: International Journal of Organic Evolution 62.8 (2008): 1965-1977.

Examples

```
library(ape)
tree<-rcoal(5)
tree<-reorder(tree,"postorder")
root<-list(y.ou.sigmsq=1 ,y.ou.root=0, x1.ou.root=0, x2.ou.root=0,x1.bm.root=0, x2.bm.root=0)
model.params<-c(0.5,0.25,0,1,0.2)
names(model.params)<-c("alpha.y","alpha.x","theta.x","sigmasq.x","tau")
reg.params<-c(0,1,1)
names(reg.params)<-c("b0","b1","b2")
ououmodel(model.params,reg.params,root=root,tree=tree)
```

---

ououprior *Draw prior samples for OUOU model*

Description

Simulate sample for parameters in ouou model given a set of hyper parameters

Usage

```
ououprior(
  prior.model.params = prior.model.params,
  prior.reg.params = prior.reg.params
)
```

Arguments

`prior.model.params`
A vectors of hyper parameters for model parameter containing (alpha.y.min, alpha.y.max) for alpha.y, (alpha.x.min, alpha.x.max) for alpha.x,(theta.y.min, theta.y.max) for theta.y, (sigmasq.x.min, sigmasq.x.max) for sigmasq.x, (tau.y.min, tau.max) for rate parameter of tau

`prior.reg.params`
A vector of hyper paramter for regression parameters. (b0.min,b0.max) for b0, (b1.min,b1.max) for b1, (b2.min,b2.max) for b2

Details

The function requires user to input hyper parameters for $\alpha_y, \alpha_x, \theta_x, \sigma_x^2, \tau$ and hyper parameters for regression parameters b_0, b_1, b_2 from uniform distribution with its minimum and maximum values.

Value

Returns the samples of model parameters and regression parameter

Examples

```
prior.model.params<-c(0,3,0,3,-5,5,0,1,0,2)
names(prior.model.params)<-c(

"alpha.y.min","alpha.y.max","alpha.x.min","alpha.x.max",

"theta.x.min","theta.x.max","sigmasq.x.min","sigmasq.x.max",

"tau.min","tau.max")
prior.reg.params<-c(-3, 3, -3, 3, -3, 3)
names(prior.reg.params)<-c("b0.min", "b0.max", "b1.min", "b1.max", "b2.min", "b2.max")
ououprior(prior.model.params=prior.model.params,prior.reg.params=prior.reg.params)
```

---

ououTrait *Parameter samples and summary statistics*

Description

Draw sample for parameters, simulate trait and compute the summary statistics for OUOU model

Usage

```
ououTrait(tree = tree, traitset = traitset, sims = sims)
```

Arguments

tree	An ape: tree object stored in phylo format
traitset	a dataframe that contains 3 traits
sims	number of trait replicate

Details

Given tree, trait sets, function `HyperParam` is called to yield the range of parameters, then function `oubmbmprior` is called to draw sample for parameter, then the function `oubmbmmodel` is applied to simulate traits through post order tree traversal algorithm, finally the summary statistics is computed by function `sumstat`.

Value

A list of vectors containing a dataframe of summary statistics, and a dataframe of parameter samples

Examples

```
## using lizard dataset (running time more > 5 sec)

data(lizard)
tree<-lizard$tree
traitset<-lizard$traitset
sims<-10
ououTrait(tree=tree,traitset=traitset,sims=sims)
```

OUprior   *Fit OU model for univariate data*

Description

Fit OU model given tree and trait

Usage

```
OUprior(tree = tree, trait = trait, model = model)
```

Arguments

`tree`	An ape: tree object stored in phylo format
`trait`	a univaraite trait
`model`	specified model preset "OU".

Details

Parameter estimates α, θ, σ^2 are estimated by BM (when $\alpha = 0$) or OU model from `geiger` for the next step analysis with function `HyperParam` to get the reasonable range of the hyper parameter as well as the ancestral value.

Value

MLE parameter estimates α, θ, σ^2.

References

1. Jhwueng, D-C. (2019) Statistical modeling for adaptive trait evolution. Under review.
2. Jhwueng, D-C., and Vasileios Maroulas. "Adaptive trait evolution in random environment." Journal of Applied Statistics 43.12 (2016): 2310-2324.
3. Hansen, Thomas F., Jason Pienaar, and Steven Hecht Orzack. "A comparative method for studying adaptation to a randomly evolving environment." Evolution: International Journal of Organic Evolution 62.8 (2008): 1965-1977.

Examples

```
library(ape)
tree<-rcoal(3)
trait<-rnorm(3)
names(trait)<-tree$tip.label
model <- "OU"
OUprior(tree=tree,trait=trait,model=model)
```

`ouqbmmodel` *Simulate traits under ouqbm model given a set of parameters*

Description

Simulate traits under ouqbm model given a set of model parameters, regression parameters, tree and ancestral values.

Usage

```
ouqbmmodel(model.params, reg.params, root = root, tree = tree)
```

Arguments

`model.params`	A vector of model parameters including alpha.y: force parameter of response trait, sigmasq.x: rate parameter of covariate and tau: rate parameter of response trait
`reg.params`	A vector of regression paramters including b0: intercept parameter of regression, b1: slope parameter of first covariate, b2: slope paramter of the second covariate
`root`	A vector of numerical values for root of species estimated from `OUprior` estimated from `OUprior`
`tree`	An ape: tree object stored in phylo format

Details

The model requires user to input model parameters $\alpha_y, \sigma_x^2, \tau$ and regression parameters b_0, b_1, b_2, a tree object, trait dataset (one response, two covariate), ancestral values(root) which is estimated by BM or OU model from `geiger`. Then the algorithm starts from the root and apply the tree traversal algorithm to simulate trait on each node according the ouqbm dynamics.

Value

Returns the trait vectors $Y = (y_1, y_2, \cdots, y_n)', X_1 = (x_{1,1}, x_{1.2}, \cdots, x_{1,n})', X_2 = (x_{2,1}, x_{2,2}, \cdots, x_{2,n})'$ simulated from the model.

References

1. Jhwueng, D. C. (2020). Modeling rate of adaptive trait evolution using Cox–Ingersoll–Ross process: An Approximate Bayesian Computation approach. Computational Statistics & Data Analysis, 145, 106924.
2. Jhwueng, D-C., and Vasileios Maroulas. "Adaptive trait evolution in random environment." Journal of Applied Statistics 43.12 (2016): 2310-2324.
3. Hansen, Thomas F., Jason Pienaar, and Steven Hecht Orzack. "A comparative method for studying adaptation to a randomly evolving environment." Evolution: International Journal of Organic Evolution 62.8 (2008): 1965-1977.

Examples

```
library(ape)
tree<-rcoal(5)
tree<-reorder(tree,"postorder")
root<-list(y.ou.sigmsq=1 ,y.ou.root=0, x1.ou.root=0, x2.ou.root=0,x1.bm.root=0, x2.bm.root=0)
model.params<-c(0.5,1,0.2)
names(model.params)<-c("alpha.y","sigmasq.x","tau")
reg.params<-c(0,1,1)
names(reg.params)<-c("b0","b1","b2")
ouqbmmodel(model.params,reg.params,root=root,tree=tree)
```

---

ouqbmprior — *Draw prior samples for ouqbm model*

Description

Simulate sample for parameters in ouqbm model given a set of hyper parameters

Usage

```
ouqbmprior(
  prior.model.params = prior.model.params,
  prior.reg.params = prior.reg.params
)
```

Arguments

`prior.model.params`
: A vectors of hyper parameters for model parameter containing (alpha.y.min, alpha.y.max) for alpha.y, (sigmasq.x.min, sigmasq.x.max) for sigmasq.x, (tau.y.min, tau.max) for rate parameter of tau

`prior.reg.params`
: A vector of hyper paramter for regression parameters. (b0.min,b0.max) for b0, (b1.min,b1.max) for b1, (b2.min,b2.max) for b2

Details

The function requires user to input hyper parameters for $\alpha_y, \sigma_x^2, \tau$ and hyper parameters for regression parameters b_0, b_1, b_2 from uniform distribution with its minimum and maximum values.

Value

Returns the samples of model parameters and regression parameter

Examples

```
prior.model.params<-c(0,3,0,3,0,1)

names(prior.model.params)<-c("alpha.y.min","alpha.y.max",

"tau.min","tau.max","sigmasq.x.min","sigmasq.x.max")

prior.reg.params<-c(-3, 3, -3, 3, -3, 3)
names(prior.reg.params)<-c("b0.min", "b0.max", "b1.min", "b1.max", "b2.min", "b2.max")
ouqbmprior(prior.model.params=prior.model.params,prior.reg.params=prior.reg.params)
```

---

ouqbmTrait *Parameter samples and summary statistics*

Description

Draw sample for parameters, simulate trait and compute the summary statistics for ouqbm model

Usage

```
ouqbmTrait(tree = tree, traitset = traitset, sims = sims)
```

Arguments

`tree`	An ape: tree object stored in phylo format
`traitset`	a dataframe that contains 3 traits
`sims`	number of trait replicate

Details

Given tree, trait sets, function `HyperParam` is called to yield the range of parameters, then function `ouqbmprior` is called to draw sample for parameter, then the function `ouqbmmodel` is applied to simulate traits through post order tree traversal algorithm, finally the summary statistics is computed by function `sumstat`.

Value

A list of vectors containing a dataframe of summary statistics, and a dataframe of parameter samples

Examples

```
## using bat dataset (running time more > 5 sec)

data(bat)
tree<-bat$tree
traitset<-bat$traitset
sims<-10
ouqbmTrait(tree=tree,traitset=traitset,sims=sims)
```

`ouqoumodel` *Simulate traits under ouqou model given a set of parameters*

Description

Simulate traits under ouqou model given a set of model parameters, regression parameters, tree and ancestral values.

Usage

```
ouqoumodel(model.params, reg.params, root = root, tree = tree)
```

Arguments

`model.params`	A vector of model parameters including alpha.y: force parameter of response trait, alpha.x: force parameter of covariate, theta.x: optimum parameter of covariate, sigmasq.x: rate parameter of covariate and tau rate parameter of response trait
`reg.params`	A vector of regression paramters including b0: intercept parameter of regression, b1: slope parameter of first covariate, b2: slope paramter of the second covariate
`root`	A vector of numerical values for root of species estimated from `OUprior`
`tree`	An ape: tree object stored in phylo format

Details

The model requires user to input model parameters $\alpha_y, \alpha_x, \theta_x, \sigma^2_x, \tau$ and regression parameters b_0, b_1, b_2, a tree object, trait dataset (one response, two covariate), ancestral values(root) which is estimated by BM or OU model from `geiger`. Then the algorithm starts from the root and apply the tree traversal algorithm to simulate trait on each node according the ouqou dynamics.

Value

Returns the trait vectors $Y = (y_1, y_2, \cdots, y_n)', X_1 = (x_{1,1}, x_{1.2}, \cdots, x_{1,n})', X_2 = (x_{2,1}, x_{2,2}, \cdots, x_{2,n})'$ simulated from the model.

References

1. Jhwueng, D-C. (2019) Statistical modeling for adaptive trait evolution. Under review.
2. Jhwueng, D-C., and Vasileios Maroulas. "Adaptive trait evolution in random environment." Journal of Applied Statistics 43.12 (2016): 2310-2324.
3. Hansen, Thomas F., Jason Pienaar, and Steven Hecht Orzack. "A comparative method for studying adaptation to a randomly evolving environment." Evolution: International Journal of Organic Evolution 62.8 (2008): 1965-1977.

Examples

```
library(ape)
tree<-rcoal(5)
tree<-reorder(tree,"postorder")
root<-list(y.ou.sigmsq=1 ,y.ou.root=0, x1.ou.root=0, x2.ou.root=0,x1.bm.root=0, x2.bm.root=0)
model.params<-c(0.5,0.25,0,1,0.2)
names(model.params)<-c("alpha.y","alpha.x","theta.x","sigmasq.x","tau")
reg.params<-c(0,1,1)
names(reg.params)<-c("b0","b1","b2")
ouqoumodel(model.params,reg.params,root=root,tree=tree)
```

---

ouqouprior *Draw prior samples for ouqou model*

Description

Simulate sample for parameters in ouqou model given a set of hyper parameters

Usage

```
ouqouprior(
  prior.model.params = prior.model.params,
  prior.reg.params = prior.reg.params
)
```

Arguments

`prior.model.params`
: A vectors of hyper parameters for model parameter containing (alpha.y.min, alpha.y.max) for alpha.y, (alpha.x.min, alpha.x.max) for alpha.x,(theta.y.min, theta.y.max) for theta.y, (sigmasq.x.min, sigmasq.x.max) for sigmasq.x, (tau.y.min, tau.max) for rate parameter of tau

`prior.reg.params`
: A vector of hyper paramter for regression parameters. (b0.min,b0.max) for b0, (b1.min,b1.max) for b1, (b2.min,b2.max) for b2

Details

The function requires user to input hyper parameters for $\alpha_y, \alpha_x, \theta_x, \sigma_x^2, \tau$ and hyper parameters for regression parameters b_0, b_1, b_2 from uniform distribution with its minimum and maximum values.

Value

Returns the samples of model parameters and regression parameter

Examples

```
prior.model.params<-c(0,3,0,3,-5,5,0,1,0,2)
names(prior.model.params)<-c(

"alpha.y.min","alpha.y.max","alpha.x.min","alpha.x.max",

"theta.x.min","theta.x.max","sigmasq.x.min","sigmasq.x.max",

"tau.min","tau.max")
prior.reg.params<-c(-3, 3, -3, 3, -3, 3)
names(prior.reg.params)<-c("b0.min", "b0.max", "b1.min", "b1.max", "b2.min", "b2.max")
ouqouprior(prior.model.params=prior.model.params,prior.reg.params=prior.reg.params)
```

---

ouqouTrait *Parameter samples and summary statistics*

Description

Draw sample for parameters, simulate trait and compute the summary statistics for ouqou model

Usage

```
ouqouTrait(tree = tree, traitset = traitset, sims = sims)
```

Arguments

`tree`	An ape: tree object stored in phylo format
`traitset`	a dataframe that contains 3 traits
`sims`	number of trait replicate

Details

Given tree, trait sets, function `HyperParam` is called to yield the range of parameters, then function `oubmbmprior` is called to draw sample for parameter, then the function `oubmbmmodel` is applied to simulate traits through post order tree traversal algorithm, finally the summary statistics is computed by function `sumstat`.

Value

A list of vectors containing a dataframe of summary statistics, and a dataframe of parameter samples

Examples

```
## using lizard dataset (running time more > 5 sec)

data(lizard)
tree<-lizard$tree
traitset<-lizard$traitset
sims<-10
ouqouTrait(tree=tree,traitset=traitset,sims=sims)
```

---

ouxy *main program to perform analysis*

Description

Analyze data and report the model estimates and model selection

Usage

```
ouxy(tree = tree, traitset = traitset, tol = 0.1, sims = 100)
```

Arguments

`tree`	An ape: tree object stored in phylo format
`traitset`	a dataframe that contains 3 traits
`tol`	acceptance rate from ABC
`sims`	number of trait replicate

Details

`ouxy` performs data analaysis under Approximate Bayesian Computation(ABC) procedure. The summary statistics for the raw traitsets are first computed by by function `sumstat`, and the parameters ranges are computed using the tree and tratisets under function `HyperParam`, and sample of prior paramters are drawn from function `oubmbmprior`, then the function `oubmbmmodel` is applied to simulate traits through post order tree traversal algorithm. The ABC procedure are then performed using sample of paramters and simulated traitset. Posterior sample are chosen using acceptance rate `sims * tol`. The posterior samples are computed using rejection method `abc` to median of the posterior samples are as reported parameter esitmate and Bayes factor is computed using function `postpr` accordingly by the ratio of the posterior model probability under each model.

Value

A list of vectors containing a dataframe of model parameter estimate, and a dataframe of Bayes factors between a pair of models

1. **table.output**: The posterior median for parameter estiamtes under each model.
2. **s.mnlog**: Bayes factor tables comparing a pair of models.

Examples

```
## using coral dataset (It takes for a whiles)

data(coral)
tree<-coral$tree
traitset<-coral$traitset
sims<-1000
output<-ouxy(tree=tree,traitset=traitset,tol=0.1,sims= sims)

## OUTPUT THE FOLLOWING
## >output$s.mnlog

## $mnlogistic
## $mnlogistic$Prob
## oubmbm    oubmcir     ououbm    ououcir
## 0.03081341 0.01533086 0.40779579 0.54605995

## $mnlogistic$BayesF
##            oubmbm     oubmcir      ououbm     ououcir
## oubmbm   1.00000000  2.00989403  0.07556087  0.05642861
## oubmcir  0.49753867  1.00000000  0.03759446  0.02807542
## ououbm  13.23436292 26.59966708  1.00000000  0.74679673
## ououcir 17.72150620 35.61834960  1.33905246  1.00000000
##
## > output$table.out
##          alpha.y alpha.x alpha.tau theta.x theta.tau   sigma.x
## OUBMBM   4.3064      NA        NA      NA        NA  7.821074
## OUOUBM   4.1240  5.2119        NA -0.5759        NA 10.117253
## OUBMCIR  4.3720      NA    4.0736      NA    1.2326  7.825912
## OUOUCIR  3.1016  4.4269    3.9930  0.0668    1.2702  9.226803
## GLS          NA      NA        NA      NA        NA        NA
##
##           tau sigma.tau        b0         b1        b2
## OUBMBM  2.2403        NA 0.1678000 0.03850000 0.2874000
## OUOUBM  2.5021        NA 0.1651000 0.03260000 0.3146000
## OUBMCIR     NA  1.492548 0.1706000 0.03760000 0.3049000
## OUOUCIR     NA  1.516047 0.1661000 0.03480000 0.2549000
## GLS         NA        NA 0.1682413 0.03931911 0.3564761
```

---

regboundfcn *range for regression parameters*

Description

Set up range for regression parameters

Usage

```
regboundfcn(olssum = olssum)
```

Arguments

`olssum` summary statistics from ordinary least square performed by `lm`.

Details

An ordinary least square analysis is performed on regression $y \sim x_1 + x_2$. Parameter estimates $\hat{b}$ and standard errors $sd(\hat{b})$ are used to construct the bound using formula $\hat{b} \pm 3sd(\hat{b})$.

Value

A vectors of values containing the range of regression parameters

Examples

```
resptrait<-rnorm(10)
predtrait1<-rnorm(10)
predtrait2<-rnorm(10)
olssum <- base::summary(lm(resptrait~predtrait1+predtrait2))
regboundfcn(olssum=olssum)
```

---

sumstat *Summary statistics*

Description

Calculate summary statistics given trait and tree

Usage

```
sumstat(trait = trait, tree = tree, ...)
```

Arguments

`trait`	A vector of numerical trait value
`tree`	An ape: tree object stored in phylo format
`...`	relevent argument

Details

This function computes the 12 summary statistics using the trait and tree. `ape` is used for computing the contrast trait from the difference between the species and its closet neighbor. For Bloomberg K and Pagel Lambda, statsitics are computed using `phytools`.

Value

Twelve summary statistcs: mean, sd, median, skewness, kurtosis from the raw data as well as from data with the difference between two closet neighbors, Bloomberg K and Pagel's lambda.

References

1. Paradis E. & Schliep K. 2018. ape 5.0: an environment for modern phylogenetics and evolutionary analyses in R. Bioinformatics 35: 526-528.
2. Blomberg, Simon P., Theodore Garland Jr, and Anthony R. Ives. "Testing for phylogenetic signal in comparative data: behavioral traits are more labile." Evolution 57.4 (2003): 717-745.
3. Pagel, Mark. "Inferring the historical patterns of biological evolution." Nature 401.6756 (1999): 877.
4. Revell, Liam J. "phytools: an R package for phylogenetic comparative biology (and other things)." Methods in Ecology and Evolution 3.2 (2012): 217-223.

Examples

```
library(ape)
tree<-rcoal(5)
trait <- rnorm(5)
names(trait)<-tree$tip.label
sumstat(trait=trait,tree=tree)
```

Index

∗Topic **datasets**
  bat, 2
  coral, 3
  lizard, 4

abc, *36*
ape, *38*

bat, 2

coral, 3

geiger, *5*, *8*, *11*, *14*, *17*, *20*, *23*, *26*, *29*, *30*, *33*

HyperParam, 3, *7*, *10*, *13*, *16*, *19*, *22*, *25*, *28*, *32*, *35*, *36*

lizard, 4
lm, *38*

oubmbmmodel, 4, *7*, *10*, *19*, *22*, *25*, *28*, *35*, *36*
oubmbmprior, 6, *7*, *10*, *19*, *22*, *25*, *28*, *35*, *36*
oubmbmTrait, *3*, 7
oubmcirmodel, 8
oubmcirprior, 9
oubmcirTrait, *3*, 10
oubmmodel, 11, *13*
oubmprior, 12, *13*
oubmTrait, 13
ougbmmodel, 14, *16*
ougbmprior, 15, *16*
ougbmTrait, 16
ougoumodel, 17
ougouprior, 18
ougouTrait, 19
ououbmmodel, 20
ououbmprior, 21
ououbmTrait, *3*, 22
ououcirmodel, 23
ououcirprior, 24
ououcirTrait, *3*, 25
ououmodel, 26
ououprior, 27
ououTrait, 28
OUprior, *3*, 29
ouqbmmodel, 30, *32*
ouqbmprior, 31, *32*
ouqbmTrait, 32
ouqoumodel, 33
ouqouprior, 34
ouqouTrait, 35
ouxy, *36*, 36

phytools, *38*
postpr, *36*

regboundfcn, *3*, 37

sumstat, *7*, *10*, *13*, *16*, *19*, *22*, *25*, *28*, *32*, *35*, *36*, 38
